# Risk Factors for Resistance to Intravenous Immunoglobulin Treatment and Coronary Artery Abnormalities in a Chinese Pediatric Population With Kawasaki Disease: A Retrospective Cohort Study

**DOI:** 10.3389/fped.2022.812644

**Published:** 2022-04-20

**Authors:** Jie Liu, Yanyun Huang, Cheng Chen, Danyan Su, Suyuan Qin, Yusheng Pang

**Affiliations:** Department of Pediatrics, First Affiliated Hospital of Guangxi Medical University, Nanning, China

**Keywords:** Kawasaki disease, high-risk, intravenous immunoglobulin resistance, coronary artery aneurysm, risk factor

## Abstract

**Background:**

The factors predicting high-risk Kawasaki disease (KD) remain unclear. Therefore, we aimed to determine the risk factors for resistance to intravenous immunoglobulin (IVIG) treatment and coronary artery aneurysm (CAA) development in a Chinese pediatric population with high-risk KD.

**Methods:**

We compared the performances of 11 scoring systems that have been reported to predict IVIG resistance among patients with KD hospitalized from January 2013 through August 2021. Patients were risk-stratified based on the optimal scoring system. The association of baseline characteristics with IVIG treatment resistance and CAA development was investigated within the high-risk group of KD.

**Results:**

In total, 346 pediatric patients with KD were included, of whom 63 (18.2%) presented with IVIG resistance. The Kobayashi score and five Chinese scoring system scores (Tang et al., Yang et al., Lan et al., Liping et al., and Wu et al.) were significantly higher in the IVIG non-responsive KD group than in the IVIG responsive KD group, and the results of the receiver operating characteristic (ROC) curves analysis were observed to be highest in the Xie Liping scoring system for IVIG resistance (area under the curve, 0.650). Especially, 87 (25.1%) patients comprised the high-risk KD group based on this optimal scoring system (≥5 points). IVIG resistance was significantly associated with the total bilirubin-to-albumin ratio (B/A ratio) [odds ratio, 7.427; 95% confidence interval (CI): 1.022–53.951]. The area under the ROC was 0.703 (95% CI: 0.586–0.821), and the cutoff point was 0.383, which indicated a sensitivity and specificity for predicting treatment resistance of 58% and 80%, respectively. The serum albumin level (odds ratio, 1.401; 95% CI: 1.049–1.869) and Z score of the left main coronary artery (odds ratio, 9.023; 95% CI: 1.070–76.112) were independent predictors of CAA development.

**Conclusions:**

In the Chinese pediatric population with KD, the Xie Liping scoring system is the most appropriate method for identifying high-risk patients, and IVIG resistance could be predicted based on the B/A ratio. Serum albumin level and Z score of the left main coronary artery at baseline were warning indicators for CAA development. More intensified or adjunctive therapies and close follow-up should be considered for high-risk patients with these risk factors.

## Introduction

Coronary artery aneurysm (CAA), the chief feature of Kawasaki disease (KD), has gradually become the most common cause of acquired cardiac disease in childhood, and the incidence of the disease has increased every year (1). Owing to the lack of specificity and dispersion of the typical symptoms, a diagnosis of KD in clinics is primarily based on an awareness of the comprehensive knowledge of the disease presentation, and KD is often missed or misdiagnosed, resulting in missing the best timing of intravenous immunoglobulin (IVIG) treatment.

Patients with no response to IVIG therapy are at a high risk of CAA development and other serious cardiovascular complications, despite receiving several rescue treatments (2–6). With an increased awareness of severe cardiovascular complications in KD, there has been considerable effort in trying to determine predictors for IVIG resistance at initial presentation in patients with KD, and multiple scoring systems based on clinical and laboratory data for predicting IVIG resistance have been developed, such as those reported by Kobayashi et al. (3), Egami et al. (7), and Sano et al. (8). Studies have discovered that patients with KD with a high Kobayashi risk score are at increased risk for IVIG resistance and may be at risk for developing a more severe coronary artery disease among the Japanese population (3). In a further investigation, adverse coronary artery outcomes have reduced from 23 to 3% by adding prednisolone to the primary IVIG treatment in high-risk KD patients with a Kobayashi risk score of ≥5 points (9). However, evidence supporting the use of the Kobayashi score for predicting IVIG resistance or CAA development in the United Kingdom and Thailand cohorts is poor (10, 11), suggesting that these scoring systems have a marked geographic diversity, which has been confirmed by studies in non-Japanese populations (12, 13).

Globally, China is among the countries with a high prevalence of KD, and many scoring systems have been developed according to the characteristics of the studied populations from various regions. Nevertheless, such scoring systems are of limited use in other regions. Likewise, data regarding the risk factors for high-risk KD are also scarce. Therefore, the present study aimed to determine the risk factors that may help clinicians identify children with high-risk KD at risk of IVIG resistance and CAA development before primary treatment among the Chinese population.

## Materials and Methods

### Study Design and Population

We retrospectively reviewed the clinical records of consecutive children with KD treated between January 2013 and August 2021 at the Pediatrics Department of First Affiliated Hospital of Guangxi Medical University, China. The criteria for the diagnosis of KD were as follows: diagnosis of KD was compliant with the Diagnostic Guidelines for Kawasaki Disease (sixth revision, issued by the Japan Kawasaki Disease Research Committee in 2020) (14), and CAA was defined as a Z score for the coronary artery internal diameter of ≥2.5 after 1 month of the disease course according to the guideline on diagnosis and management of cardiovascular sequelae in Kawasaki disease (JCS/JSCS 2020) (15). Children were excluded, if treatment after the first 10 days of the onset of fever and the diagnosis of KD were not clear, if they received IVIG or corticosteroid (e.g., methylprednisolone or prednisone) therapy outside the hospital, or had incomplete data required for statistical analyses.

All patients received initial IVIG (2 g/kg per 24 h) and oral aspirin (30–50 mg/kg per day) until resolution of their fever for 72 h at least. Then, they received aspirin (3–5 mg/kg per day) for 2 months from disease onset. Patients with IVIG resistance, defined as having persistent or recrudescent fever (temperature, >38.0°C) for at least 36 h but not longer than 7 days after completion of the initial IVIG infusion (2 g/kg), received a second dose of gamma globulin therapy (2 g/kg). Corticosteroid (methylprednisolone was administered by intravenous injection at a dose of 2 mg/kg per day, twice a day. Afterwards, the administration was tapered and withdrawn until the normalization of the C-reactive protein (CRP) levels, and oral prednisolone administration was initiated from 2 mg/kg and reduced to 1 mg/kg and finally to 0.5 mg/kg per day and tapered for ≥2 weeks) was administered due to unremitting fever after completion of the second IVIG treatment. According to their responsiveness to the initial IVIG treatment, all children were divided into two groups as follows: the IVIG responsive (*n* = 283) and IVIG non-responsive KD groups (*n* = 63).

As is well known, the focus of clinical attention has been on how to screen patients with a high risk of IVIG resistance and administer intensive therapy. Currently, there is no universal risk scoring system that applies to all populations. Based on this, differences in 11 existing scoring systems (3, 7, 8, 12, 16–22) that have been previously reported to identify patients with KD at high risk of IVIG resistance were compared between the study groups. Patients were assigned scores according to the rules of each scoring system (see Supplementary File 1). Eligible patients were diagnosed with high-risk KD using the optimal scoring system and the association of baseline characteristics with IVIG treatment resistance and CAA development was investigated within the high-risk group of KD. To further verify our results, we analyzed high-risk patients with KD based on the Kobayashi score, which is widely used in Japanese patients, using the same assay (see Supplementary File 2).

### Data Collection

The following clinical and laboratory data collected from the medical charts of the patients enrolled in this study were reviewed using a standardized form: (i) general demographic data: age, sex, height, weight, and body mass index (BMI); (ii) clinical manifestations: duration of fever before admission, pediatric sequential organ failure assessment (pSOFA) score on the day of admission, illness day at treatment (illness day 1 = 1st day of fever), response to IVIG therapy, incidence of incomplete KD and CAA, and total score based on the optimal scoring system; and (iii) laboratory indicators: the highest value was selected for analysis in the case of the white blood cell count (WBC), neutrophil count, aspartate aminotransferase (AST) level, alanine aminotransferase (ALT) level, total bilirubin (TSB) level, and CRP level, whereas the lowest value was selected in the case of the lymphocyte count, hemoglobin concentration, platelet (PLT) count, serum albumin (ALB) concentration, and serum sodium concentration. All the laboratory indicators were collected for assessment at the time during the acute febrile period and before the initial IVIG treatment. We calculated the neutrophil-to-lymphocyte count ratio, platelet-to-lymphocyte count ratio, TSB-to-ALB ratio (B/A ratio), and capillary leakage index based on the aforementioned indicators. We also collected data on the coronary arterial internal diameters of the right coronary artery, left main coronary artery, left anterior descending artery, and left circumflex coronary artery from echocardiography that was performed once a week within 2 months after disease onset, and the Z score of the coronary artery corrected for the body surface area was determined and recorded (iv).

### Statistical Analyses

Normality of distribution was verified using the Shapiro–Wilk and homogeneity tests. Measurement data with a normal distribution are expressed as means ± standard deviations, and the two-independent sample t-test was used to compare such data between the groups. Measurement data that did not have a normal distribution are expressed as medians (four-digit interval) [P_50_ (P_25_, P_75_)], and these data were compared between the groups using the Mann–Whitney U test. Enumeration data are expressed as a percentage (%). The chi-square or Pearson's chi-square test was used to perform intergroup comparisons. Significant indices were analyzed using multivariate logistic regression analysis to determine the risk factors, and the best threshold for the significant parameter was constructed using receiver operating characteristic (ROC) curves. The *P*-values were two-tailed with *P* <0.05 considered statistically significant. Statistical analyses were performed using SPSS, version 26.0 (IBM Corp., Armonk, NY, USA).

## Results

### Baseline Characteristics

Over the period of observation, 452 children were admitted to the Pediatrics of First Affiliated Hospital of Guangxi Medical University with a diagnosis of KD. In total, 106 children were excluded from this study, including 53 children who were diagnosed after 10 days of illness, 17 children who received IVIG or corticosteroid therapy outside the hospital, and 36 with incomplete clinical or laboratory data. Ultimately, 346 children were included in this study. Of them, 63 (18.2%) children had initial IVIG resistance, and 16 (25.4%) of those children received corticosteroid therapy in addition to the second dose of gamma globulin therapy. Afterwards their symptoms alleviated and no other biologic agents, such as infliximab, cyclosporine, anakinra, cyclophosphamide, or plasma exchange, were administered during this period. The mean age of the IVIG non-responsive KD group was 30 months (range, 2–152 months) with a male-to-female ratio of 2.7:1 (46 boys and 17 girls); there were 27 cases (42.9%) of incomplete KD and 19 cases (30.2%) of CAA. The mean age of the IVIG responsive KD group was 28 months (range, 2–158 months) with a male-to-female ratio of 2.3:1 (198 boys and 85 girls); there were 117 cases (41.3%) of incomplete KD and 69 cases (24.4%) of CAA. There were no disease recurrences or deaths in either group.

### Comparisons of the Scoring Systems

**Figure 1:**
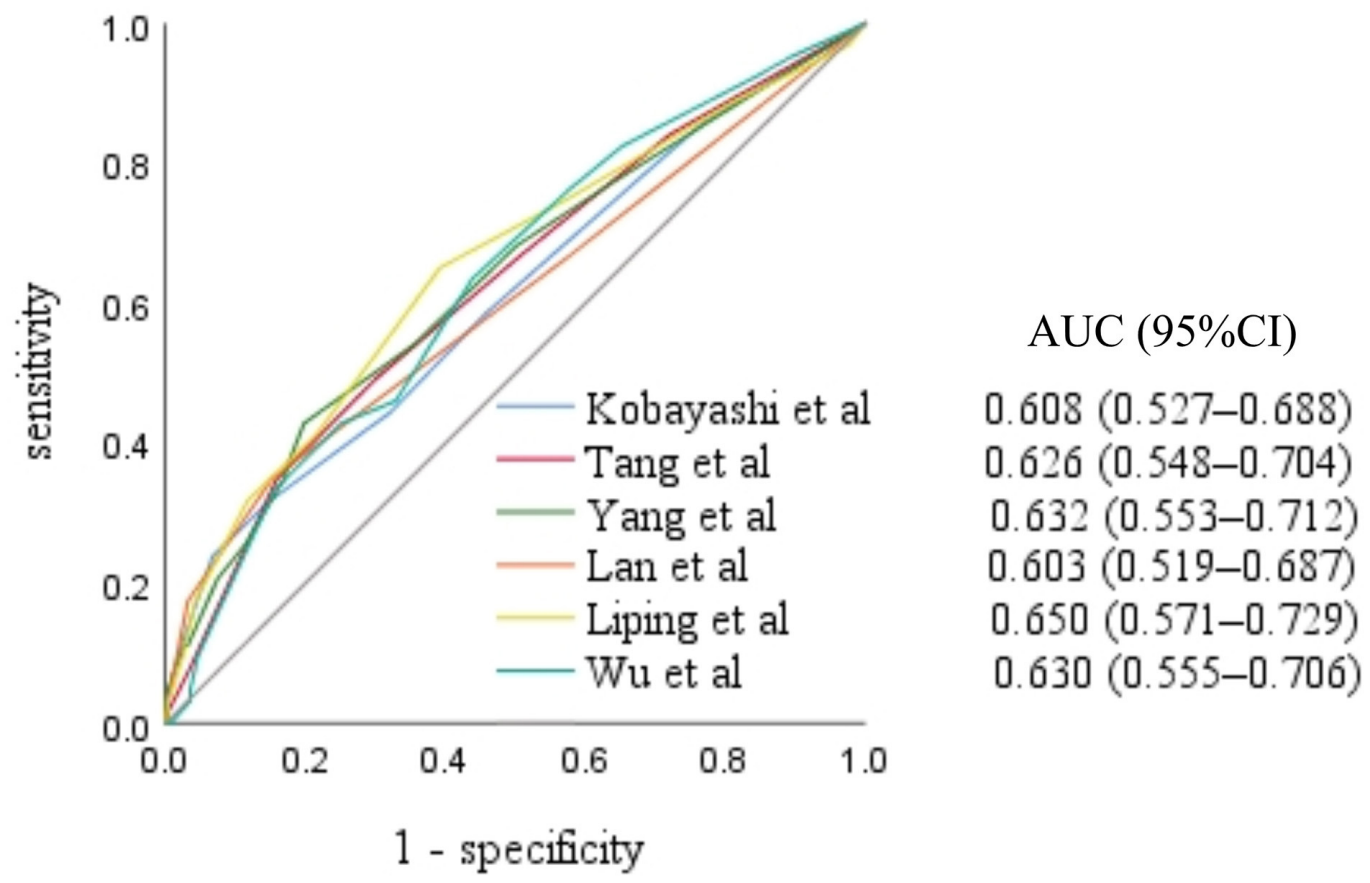
Receiver operating characteristic curves for the scoring systems scores in the intravenous immunoglobulin non-responsive Kawasaki disease group. AUC, area under the curve; CI, confidence interval.

Results of the analysis of the 11 scoring systems by study group are presented in the Supplementary File 1. The Kobayashi score (3) and five Chinese scoring system scores (12, 18, 20–22) were higher in the IVIG non-responsive KD group than in the IVIG responsive KD group, and the differences were statistically significant (all, *P* <0.05). The area under the ROC curve for the Xie Liping risk scoring system was the largest, with an area of 0.650 [95% confidence interval (CI): 0.571–0.729], while the Kobayashi score had an area of 0.608 (95% CI: 0.527–0.688) (Figure 1).

### Intragroup Comparisons Between the High-Risk KD Groups to Analyze the Risk Factors for High-Risk KD

#### Analysis of Risk Factors for IVIG Resistance in High-Risk KD

##### Comparisons of the Clinical Characteristics

Eighty-seven hospitalized children were diagnosed with high-risk KD according to the optimal scoring system (Xie Liping risk score of ≥ 5 points). Of these, 26 patients with IVIG resistance were enrolled in the IVIG non-responsive sub-group, and eight (30.8%) of them had used corticosteroid therapy in addition to the second dose of gamma globulin therapy. The remaining 61 patients were enrolled in the IVIG responsive sub-group. The sum of scores based on the Xie Liping risk scoring system, and the proportion of days of illness at primary treatment (<4 or 5 days), were higher in the IVIG non-responsive sub-group than in the IVIG responsive sub-group with statistically significant differences (both, *P* < 0.05). However, there were no statistically significant differences between the sub-groups with respect to age, sex, height, weight, BMI, pSOFA score, fever duration before admission, and the incidence of incomplete KD or CAA (all, *P* > 0.05). In terms of laboratory indicators, the IVIG non-responsive sub-group showed higher values of the serum ALT, AST, TSB, and B/A ratio and lower values of the WBC and serum sodium than those of the IVIG responsive sub-group, and the differences were statistically significant (all, *P* < 0.05), as shown in Table 1.

**Table 1:** Comparison of the baseline characteristics between the intravenous immunoglobulin non-responsive and responsive sub-groups.

	**Total (*n* = 87)**	**IVIG non-responsive sub-group (*n* = 26)**	**IVIG responsive sub-group (*n* = 61)**	***P*-value**
Age [month, *P*_50_ (*P*_25_, *P*_75_)]	31.00 (20.00, 51.00)	28.50 (13.75, 63.25)	31.00 (20.00, 44.50)	0.721
<1 year [*n* (%)]	9 (10.3)	4 (15.4)	5 (8.2)	0.533
Male [*n* (%)]	66 (75.9)	20 (76.9)	46 (75.4)	0.880
Height [m, *P*_50_ (*P*_25_, *P*_75_)]	0.92 (0.78, 1.05)	0.92 (0.77, 1.11)	0.92 (0.81, 1.03)	0.623
Weight [kg, *P*_50_ (*P*_25_, *P*_75_)]	13.00 (10.00, 16.00)	13.50 (9.35, 17.55)	12.80 (10.00, 15.50)	0.478
BMI [kg/m^2^, *P*_50_ (*P*_25_, *P*_75_)]	15.33 (14.61, 16.60)	15.88 (14.51, 17.30)	15.24 (14.65, 16.39)	0.173
pSOFA score (point, mean ± SD)	0.70 ± 1.52	1.12 ± 2.16	0.52 ± 1.12	0.097
Fever duration before admission (day, mean ± SD)	6.29 ± 3.04	6.58 ± 3.60	6.16 ± 2.79	0.565
Days of illness at primary treatment (day, mean ± SD)	6.84 ± 3.48	5.96 ± 3.86	7.21 ± 3.26	0.125
≤ 4 days [*n* (%)]	19 (21.8)	10 (38.5)	9 (14.8)	0.014
≤ 5 days [*n* (%)]	42 (48.3)	17 (65.4)	25 (41.0)	0.037
Incomplete KD [*n* (%)]	50 (57.5)	16 (61.5)	34 (55.7)	0.616
CAA [*n* (%)]	18 (20.7)	7 (26.9)	11 (18.0)	0.349
Score (12) (point, mean ± SD)	6.06 ± 1.13	6.50 ± 1.24	5.87 ± 1.04	0.017
White blood cell count ( ×10^9^/L, ref. 5–12 ×10^9^/L, mean ± SD)	15.40 ± 7.03	12.96 ± 6.59	16.44 ± 7.01	0.034
Neutrophils count ( ×10^9^/L, ref. 1.8–6.3 ×10^9^/L, mean ± SD)	11.36 ± 6.39	10.00 ± 5.73	11.93 ± 6.62	0.197
NLR [*P*_50_ (*P*_25_, *P*_75_)]	4.94 (2.38, 8.00)	5.52 (2.61, 11.35)	4.66 (2.32, 7.36)	0.209
Hemoglobin (g/L, ref. 120–160 g/L, mean ± SD)	101.69 ± 12.67	104.57 ± 14.80	100.47 ± 11.56	0.168
Platelet count ( ×10^12^/L, ref. 125–350 ×10^9^/L, mean ± SD)	340.13 ± 155.03	318.55 ± 187.88	349.33 ± 139.48	0.400
PLR [*P*_50_ (*P*_25_, *P*_75_)]	125.00 (83.33,250.00)	225.00 (75.80, 333.33)	125.00 (83.33, 200.00)	0.080
CRP [mg/L, ref. 0–10 mg/L, *P*_50_ (*P*_25_, *P*_75_)]	97.20 (40.74, 180.63)	98.40 (53.13, 145.28)	97.12 (39.49, 192.00)	0.989
Sodium [mmol/L, ref. 137–147 mmol/L, mean ± SD]	134.66 ± 3.79	132.98 ± 3.55	135.37 ± 3.70	0.007
≤ 133 mmol/L [n(%)]	32 (36.8)	14 (53.8)	18 (29.5)	0.031
ALT [U/L, ref. 7–45 U/L, mean ± SD]	67.95 ± 61.34	93.10 ± 60.28	57.23 ± 59.06	0.012
AST [U/L, ref. 13–40 U/L, *P*_50_ (*P*_25_, *P*_75_)]	32.00 (24.00, 46.00)	46.60 (31.50, 95.50)	30.00 (22.00, 37.50)	<0.001
Total bilirubin [μmol/L, ref. 3.4–20.5 μmol/L, *P*_50_ (*P*_25_, *P*_75_)]	7.40 (4.00, 15.70)	13.80 (5.80, 20.90)	6.60 (3.45, 11.65)	0.007
Albumin (g/L, ref. 40–55 g/L, mean ± SD)	31.79 ± 4.33	30.90 ± 4.75	32.16 ± 4.13	0.217
≤ 34 g/L [*n* (%)]	67 (77.0)	20 (76.9)	47 (77.0)	0.990
B/A ratio [*P*_50_ (*P*_25_, *P*_75_)]	0.24 (0.12, 0.53)	0.39 (0.22, 0.73)	0.21 (0.11, 0.36)	0.003
CLI [*P*_50_ (*P*_25_, *P*_75_)]	3.11 (1.28, 5.13)	3.22 (1.72, 4.57)	3.10 (1.22, 5.33)	0.897

*IVIG, intravenous immunoglobulin; BMI, body mass index; pSOFA, pediatric sequential organ failure assessment; KD, Kawasaki disease; CAA, coronary artery aneurysm; NLR, neutrophil-to-lymphocyte count ratio; PLR, platelet-to-lymphocyte count ratio; CRP, C-reactive protein; ALT, alanine aminotransferase; AST, aspartate aminotransferase; B/A ratio, total bilirubin-to-albumin ratio; CLI, capillary leakage index*.

##### Results of the Multi-Factor Logistic Analysis

**Figure 2:**
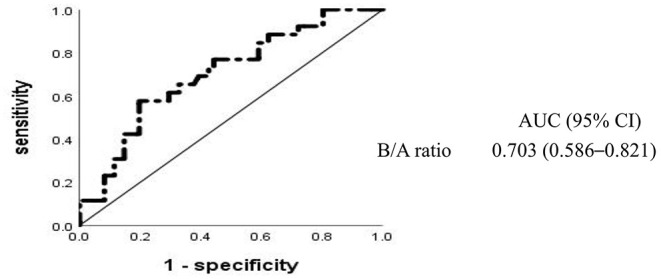
Receiver operating characteristic curves for intravenous immunoglobulin resistance in high-risk Kawasaki disease. AUC, area under the curve; CI, confidence interval; B/A, total bilirubin-to-albumin ratio.

To determine the relative effect of each risk factor for IVIG resistance in high-risk KD, we performed a logistic regression analysis, which revealed that high-risk KD with IVIG resistance was significantly associated with six baseline laboratory variables (WBC, serum levels of ALT, AST, TSB, and sodium, and B/A ratio) and two clinical characteristics (days of illness at primary treatment and the Xie Liping risk score). These variables plus the CRP level, PLT count, and age <1 year, all of which were previously reported as risk factors for IVIG resistance (3, 7, 16, 22, 23), but not the Xie Liping risk score or TSB level, were included in the logistic regression analysis (Table 2). The B/A ratio, calculated as the TSB level divided by the ALB level, was included in the multivariable analysis instead of the two separate indicators; the Xie Liping risk score was also excluded from the multivariable analysis because the variables included in this risk score, such as days of illness at primary treatment, the serum sodium level, age, the WBC count instead of the percentage of neutrophils, and B/A ratio instead of the ALB level, were included in the multivariable model. The B/A ratio was the only significant independent predictor of IVIG resistance in high-risk KD cases. The area under the ROC curve for the B/A ratio was 0.703 (95% CI: 0.586–0.821) (Figure 2), and the sensitivity and specificity for predicting IVIG resistance in high-risk KD patients were 58% and 80%, respectively, at a cutoff point of 0.383 (Table 3). Similarly, the B/A ratio was observed to be a significant predictor of IVIG resistance in high-risk patients with KD based on the Kobayashi score [OR: 10.336 (95% CI: 1.240–86.126), *P* = 0.031].

**Table 2:** Results of logistic regression analyses of intravenous immunoglobulin resistance.

**Characteristics**	**Univariable**		**Multivariable**	
	**Odds ratio (95% CI)**	***P*-value**	**Odds ratio (95% CI)**	***P*-value**
White blood cell count	1.083 (1.004–1.168)	0.038	1.087 (0.972–1.215)	0.157
Sodium	1.199 (1.045–1.376)	0.010	1.099 (0.938–1.285)	0.243
ALT	1.009 (1.001–1.018)	0.020	1.001 (0.989–1.013)	0.887
AST	1.025 (1.009–1.040)	0.002	1.015 (0.992–1.038)	0.206
B/A ratio	3.069 (1.126–8.364)	0.028	7.427 (1.022–53.951)	0.047
Days of illness at primary treatment	1.136 (0.962–1.342)	0.133	1.245 (0.983–1.580)	0.069
CRP	0.999 (0.992–1.006)	0.786	0.995 (0.985–1.005)	0.313
Platelet count	1.001 (0.998–1.004)	0.396	1.004 (0.999–1.009)	0.135
<1 year	0.491 (0.121–2.000)	0.321	0.479 (0.093–2.452)	0.377

*CI, confidence interval; ALT, alanine aminotransferase; AST, aspartate aminotransferase; B/A ratio, total bilirubin-to-albumin ratio; CRP, C-reactive protein*.

**Table 3:** Baseline B/A ratios for predicting high-risk Kawasaki disease.

**Baseline B/A ratio**	**Sensitivity, %**	**Specificity, %**	**PPV, %**	**NPV, %**
≥0.383	58	80	55.6	81.7
≥0.373	58	79	53.6	81.4
≥0.361	58	77	51.7	81.0
≥0.387	54	80	53.8	80.3
≥0.357	58	75	50.0	80.7

*B/A ratio, total bilirubin-to-albumin ratio; PPV, positive predictive value; NPV, negative predictive value*.

#### Analysis of Risk Factors for CAA in High-Risk KD

##### Comparisons of the Clinical Characteristics

Although there were 87 patients in the high-risk group, only 18 (20.7%) of these patients had CAA, and there were no statistically significant differences between the sub-groups with respect to the demographic and clinical manifestations (all, *P* > 0.05). The univariable analysis identified only one laboratory index, the serum ALB level, which, together with the Z score of the coronary artery internal diameter in the acute phase of high-risk KD, was significantly associated with CAA development (Table 4). Multivariable analysis revealed that the serum ALB level and the Z score of the left main coronary artery internal diameter were significant independent predictors of CAA development, and this difference remained significant when corrected for age and sex (Table 5). In high-risk patients with KD based on the Kobayashi score, the Z-score of the left main coronary artery internal diameter was also significantly associated with CAA development [OR: 19.097 (95% CI: 1.871–194.902), *P* = 0.013].

**Table 4:** Comparisons of the baseline characteristics between the coronary artery aneurysm and non-coronary artery aneurysm sub-groups.

	**Total (*n* = 87)**	**CAA sub-group (*n* = 18)**	**Non-CAA sub-group (*n* = 69)**	***P*-value**
Age (month, mean± SD)	36.49 ± 25.86	35.44 ± 36.71	36.77 ± 22.54	0.848
<1 year [*n* (%)]	9 (10.3)	4 (22.2)	5 (7.2)	0.155
Male [*n* (%)]	66 (75.9)	14 (77.8)	52 (75.4)	1.000
Height (m, mean ± SD)	0.93 ± 0.18	0.90 ± 0.25	0.94 ± 0.16	0.503
Weight (kg, mean ± SD)	13.92 ± 5.84	13.85 ± 8.26	13.94 ± 5.10	0.952
BMI (kg/m^2^, mean ± SD)	15.70 ± 1.65	16.26 ± 1.67	15.55 ± 1.63	0.107
pSOFA score [point, *P*_50_ (*P*_25_, *P*_75_)]	0.00 (0.00, 1.00)	0.50 (0.00, 2.00)	0.00 (0.00, 1.00)	0.102
Fever duration before admission (day, mean ± SD)	6.29 ± 3.04	6.72 ± 3.66	6.17 ± 2.88	0.499
Days of illness at primary treatment (day, mean ± SD)	6.84 ± 3.48	7.72 ± 4.56	6.61 ± 3.14	0.228
≤ 4 days [*n* (%)]	19 (21.8)	5 (27.8)	14 (20.3)	0.716
≤ 5 days [*n* (%)]	42 (48.3)	6 (33.3)	36 (52.2)	0.154
Incomplete KD [*n* (%)]	50 (57.5)	8 (44.4)	42 (60.9)	0.209
IVIG resistance [*n* (%)]	26 (29.9)	7 (38.9)	19 (27.5)	0.349
Score (12) [point, mean ± SD]	6.06 ± 1.13	6.17 ± 1.20	6.03 ± 1.12	0.649
White blood cell count [ ×10^9^/L, ref. 5–12 ×10^9^/L, *P*_50_ (*P*_25_, *P*_75_)]	14.47 (10.75, 20.05)	18.77 (8.80, 23.69)	13.95 (11.27, 18.71)	0.245
Neutrophils count ( ×10^9^/L, ref.1.8–6.3 ×10^9^/L, mean ± SD)	11.36 ± 6.39	13.90 ± 7.77	10.69 ± 5.87	0.058
NLR (mean ± SD)	5.70 ± 4.35	6.42 ± 3.67	5.52 ± 4.52	0.438
Hemoglobin (g/L, ref. 120–160 g/L, mean ± SD)	101.69 ± 12.67	97.40 ± 14.15	102.81 ± 12.11	0.107
Platelet count ( ×10^12^/L, ref. 125–350 ×10^9^/L, mean ± SD)	340.13 ± 155.03	310.91 ± 180.20	347.75 ± 148.31	0.372
PLR (mean ± SD)	168.44 ± 114.75	145.95 ± 104.35	174.31 ± 117.31	0.353
CRP (mg/L, ref. 0–10 mg/L, mean ± SD)	105.39 ± 65.04	118.62 ± 65.74	101.94 ± 64.89	0.335
Sodium (mmol/L, ref. 137–147 mmol/L, mean ± SD)	134.66 ± 3.79	134.21 ± 4.09	134.77 ± 3.73	0.579
≤ 133 mmol/L [*n* (%)]	32 (36.8)	7 (38.9)	25 (36.2)	0.835
ALT [U/L, ref. 7–45 U/L, *P*_50_ (*P*_25_, *P*_75_)]	49.00 (21.00, 96.00)	62.00 (30.50, 93.00)	43.00 (18.50,108.00)	0.335
AST (U/L, ref. 13–40 U/L, mean ± SD)	44.44 ± 32.89	40.72 ± 26.22	45.41 ± 34.52	0.593
Total bilirubin (μmol/L, ref. 3.4–20.5 μmol/L, mean ± SD)	13.24 ± 16.27	16.74 ± 17.15	12.32 ± 16.03	0.307
Albumin (g/L, ref. 40–55 g/L, mean ± SD)	31.79 ± 4.33	29.13 ± 3.53	32.48 ± 4.27	0.003
≤ 34 g/L [*n* (%)]	67 (77.0)	16 (88.9)	51 (73.9)	0.303
B/A ratio (mean ± SD)	0.43 ± 0.57	0.60 ± 0.68	0.38 ± 0.53	0.157
CLI (mean ± SD)	3.34 ± 2.07	4.06 ± 2.22	3.16 ± 2.01	0.100
*Z* score of coronary artery internal diameter				
Left main coronary artery (mean ± SD)	2.34 ± 1.09	3.69 ± 0.92	1.99 ± 0.82	<0.001
Right coronary artery (mean ± SD)	2.03 ± 1.38	3.23 ± 1.32	1.72 ± 1.22	<0.001
Baseline maximum *Z* score (mean ± SD)	2.65 ± 1.13	4.01 ± 0.79	2.30 ± 0.93	<0.001

*CAA, coronary artery aneurysm; BMI, body mass index; pSOFA, pediatric sequential organ failure assessment; KD, Kawasaki disease; IVIG, intravenous immunoglobulin; NLR, neutrophil-to-lymphocyte count ratio; PLR, platelet-to-lymphocyte count ratio; CRP, C-reactive protein; ALT, alanine aminotransferase; AST, aspartate aminotransferase; B/A ratio, total bilirubin-to-albumin ratio; CLI, capillary leakage index*.

**Table 5:** Results of logistic regression analyses of coronary artery aneurysm.

**Characteristic**	**Univariable**		**Multivariable**		**Adjust** ^*****#*****^	
	**Odds ratio (95% CI)**	***P*-value**	**Odds ratio (95% CI)**	***P*-value**	**Odds ratio (95% CI)**	***P*-value**
Albumin	1.255(1.071–1.468)	0.005	1.361 (1.053–1.757)	0.019	1.401 (1.049–1.869)	0.022
*Z* score of left main coronary artery	9.505(3.266–27.669)	<0.001	8.025 (1.062–60.620)	0.044	9.023 (1.070–76.112)	0.043
*Z* score of right coronary artery	2.654 (1.576–4.469)	<0.001	1.232 (0.490–3.101)	0.657	1.206 (0.466–3.122)	0.699
Baseline maximum *Z* score	7.250 (2.882–18.239)	<0.001	1.632 (0.162–16.460)	0.678	1.734 (0.154–19.518)	0.656

*# Indicates a significant relationship after correction for age and sex; CI, confidence interval*.

##### ROC Analysis of the Risk Factors for CAA

**Figure 3:**
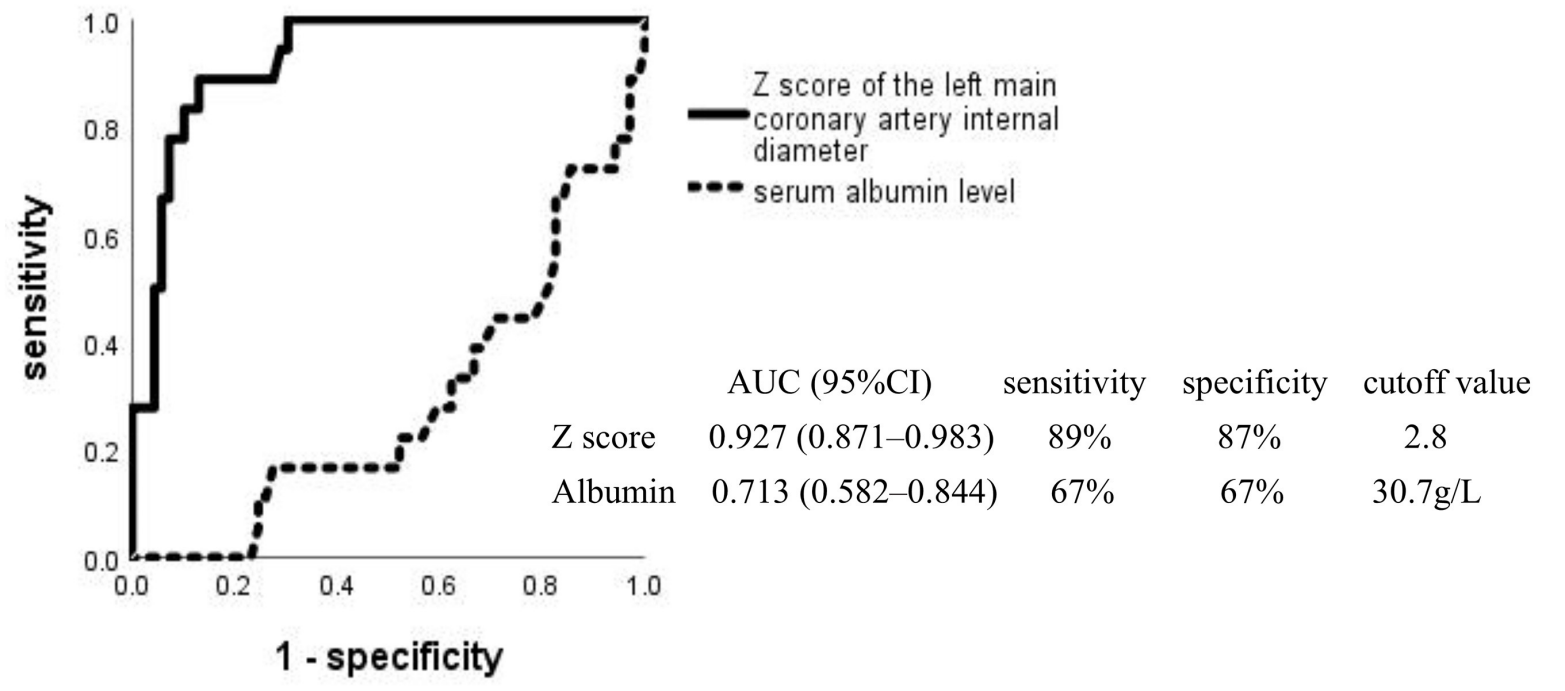
Receiver operating characteristic curves for coronary artery aneurysm development in high-risk Kawasaki disease. AUC, area under the curve; CI, confidence interval.

**Figure 4:**
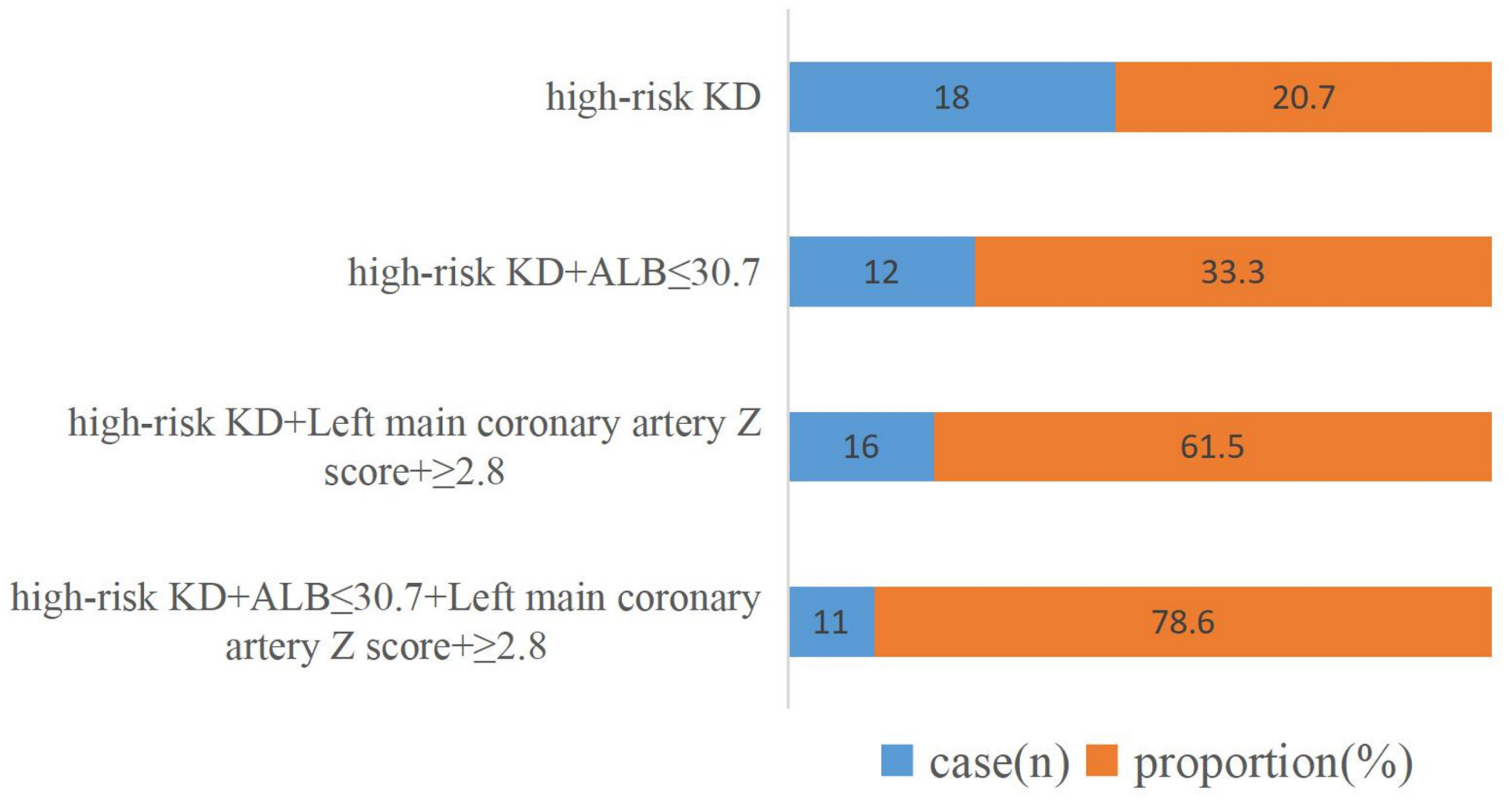
Incidence of coronary artery abnormalities after disease onset per risk factor. KD, Kawasaki disease; ALB, serum albumin level.

Multivariate logistic regression analysis showed that a low level of serum ALB and a high Z score of the left main coronary artery internal diameter were significantly associated with CAA. The respective areas under the ROC curve for the serum ALB level and Z score of the left main coronary artery internal diameter were 0.713 and 0.927, at cutoff points of 30.7 g/L and 2.8 (Figure 3). Figure 4 shows the incidence of CAA in high-risk patients by risk factor and demonstrates that the risk of CAA development increased with an increase in the number of risk factors.

## Discussion

This study identified risk factors for resistance to IVIG treatment and CAA development in a Chinese pediatric population with high-risk KD. In addition, we observed that in our patient group with KD, the Xie Liping risk score (12) had potential value in predicting IVIG resistance, and the B/A ratio alone was highly associated with IVIG resistance in high-risk patients with a score ≥ 5 points. Unexpectedly, the obtained results were revealed to be similar for the high-risk patients with KD as defined by a Kobayashi score ≥4 points (see Supplementary File 2). TSB, which is the numerator in the equation for the B/A ratio, is positively associated with IVIG resistance, whereas the serum ALB level, which is the denominator, is negatively associated with IVIG resistance; both these variables are reported to predict IVIG resistance (8, 12, 14, 19, 20, 22). However, our findings were not entirely consistent with the findings of the aforementioned studies. We observed that the B/A ratio was the only risk factor associated with IVIG resistance. In conjunction with those reported in the literature, our results may indicate that an isolated indicator is of limited utility in predicting IVIG resistance for high-risk patients with KD; thus, a composite index that incorporates some laboratory indicators may be of greater value. To the best of our knowledge, the present study is the first to suggest that a high B/A ratio is a risk factor for predicting IVIG resistance in high-risk patients with KD, although future large-scale studies are needed to verify this finding.

The present study discovered that a low serum ALB level and a high Z score of the left main coronary artery internal diameter at baseline were independently associated with CAA development in high-risk patients with KD, which was consistent with the findings of a study regarding the risk factors for coronary aneurysms and progressive coronary dilatation in Taiwanese patients (24). Hepatic dysfunction as a common complication during the acute KD episode has been evidenced in past studies (25), in which hypoalbuminemia was the most common type. The serum ALB level is also one of the variables included in the Harada score (26) in Japan, which has a cutoff <35 g/L and was designed to predict CAA development. The possible mechanisms of hypoalbuminemia in CAA secondary to KD are related to the capillary permeability associated with systemic vasculitis (27), and the present study also observed a higher capillary leakage index in the CAA sub-group compared with that in the non-CAA sub-group. However, the difference failed to reach statistical significance, suggesting that high-risk KD patients with higher inflammatory reactions and stronger immune responses have a higher leakage of serum ALB, which may be a characteristic of high-risk patients with CAA, indicating that it is important to follow these patients carefully.

Coronary artery lesions have always been a popular research topic in KD, and the extent of coronary artery expansion during the acute phase of the disease has a negative correlation with the chance for them to return to normal; giant coronary artery aneurysms do not revert to a normal morphology (28–30). Several studies have reported that the likelihood of coronary artery regression within the normal range depends on the size of the baseline Z score of the coronary artery (31, 32). The results obtained in the current study are comparable to those of the previous one. Our study observed that the baseline Z score of the left main coronary artery before the primary treatment was a heightened risk factor for CAA development, and the same result was observed in the high-risk patients with KD as defined by a Kobayashi score ≥4 points (see Supplementary File 2). However, inconsistent with the results of a multicenter and prospective observational study that was conducted in Japan, our study showed no relationships between age <1 year or IVIG resistance and CAA development (23), which was probably because of the small sample size of high-risk patients with KD. Our study may have been underpowered to conduct a highly informative multivariate analysis. Nevertheless, the binary logistic regression analysis also revealed that a high Z score of the left main coronary artery was highly significantly related to a higher likelihood of CAA development at 1 month of illness in high-risk patients with KD. Even after correction for age and sex, our results remained significant, suggesting that close monitoring with frequent echocardiography is needed for high-risk patients with these risk factors.

Our study has some limitations. First, the sample size of this study limited the power of the analyses; hence, studies with larger sample sizes are warranted. Second, because of non-routine testing, some indicators that have been reported to be associated with IVIG resistance or CAA were not considered in the study design, e.g., the levels of serum procalcitonin, interleukin-6, and brain natriuretic peptide. Third, the collection of limited laboratory data precluded the testing of other scoring systems, and the Xie Liping risk score, which was developed in China to predict IVIG resistance, has not been adapted to other ethnicities, and high-risk patients with KD, as defined by this scoring system, may not be generalizable to other populations. However, the results obtained for high-risk patients with KD, as defined by the Kobayashi score, were mostly similar. Since all the participants in our study were Chinese, the Xie Liping risk score was appropriate. Hence, more prospective studies are needed to validate a more valuable risk scoring system for identifying children with high-risk KD.

## Conclusions

In the Chinese pediatric population with KD, the Xie Liping scoring system is the most appropriate method for identifying high-risk patients, and IVIG resistance could be predicted based on the B/A ratio. Serum ALB level and Z score of the left main coronary artery at baseline were risk factors significantly associated with CAA development. To reduce CAA development in high-risk patients, more intensified or adjunctive therapies should be considered, and intensive monitoring with echocardiography should be required. Future studies are needed to verify the association of these risk factors in different ethnic populations.

## Data Availability Statement

The original contributions presented in the study are included in the article/Supplementary Material, further inquiries can be directed to the corresponding author.

## Ethics Statement

The approval for this research was obtained from the Medical Ethics Committee of the First Affiliated Hospital of GuangXi Medical University [Code Number: 2021(KY-E-240)] and informed consent was obtained from the parents of each participant.

## Author Contributions

JL, YH, DS, and YP: conceptualization. JL, CC, and SQ: formal analysis. DS and SQ: methodology. JL and YH: writing—original draft. JL and YP: writing—review and editing. All authors approved the final manuscript to be submitted and agreed to be accountable for all aspects of the work.

## Conflict of Interest

The authors declare that the research was conducted in the absence of any commercial or financial relationships that could be construed as a potential conflict of interest.

## Publisher's Note

All claims expressed in this article are solely those of the authors and do not necessarily represent those of their affiliated organizations, or those of the publisher, the editors and the reviewers. Any product that may be evaluated in this article, or claim that may be made by its manufacturer, is not guaranteed or endorsed by the publisher.
