# Supplementary material for: Risk Factors for Resistance to Intravenous Immunoglobulin Treatment and Coronary Artery Abnormalities in a Chinese Pediatric Population With Kawasaki Disease: A Retrospective Cohort Study
Source: Front Pediatr. 2022 Apr 20;10:812644. doi: 10.3389/fped.2022.812644 (PMC9067160; doi:10.3389/fped.2022.812644)
Supplement: Supplementary file 1 [file Data_Sheet_1.pdf]

Early warning score systems for predicting intravenous immunoglobulin resistance in Kawasaki disease

Comparisons of the scoring systems

Eleven existing scoring systems that have been previously reported to identify patients with Kawasaki disease(KD) at high risk of intravenous immunoglobulin (IVIG) resistance included 4 from Japan, 6 from the mainland of China and 1 from Taiwan. Kobayashi, Egami, Sano and Kawamura scoring system from Japan with sensitivity range 71% to 86%, specificity range 68% to 86%; the other 6 scoring systems from the mainland of China with sensitivity range 54% to 91%, specificity range 61% to 81%; and Formosa scoring system from Taiwan with 91% specificity and 81% sensitivity, respectively. Detailed the rules of each scoring system is listed in Table 1. Results of the analysis of the 11 scoring systems by our study group are shown in Table 2. The Kobayashi score (1) and five Chinese scoring system scores (Tang et al. (7), Yang et al. (8), Lan et al. (9), Liping et al. (10), and Wu et al. (11)) were higher in the IVIG non-responsive KD group than in the IVIG responsive KD group, and the differences were statistically significant (all, $P<0.05$).

Table 1 Rules of the eleven existing scoring systems

Prediction models	Nation	Year	Enrolled patients (n)	Patients with IVIG resistance (n)	Risk factors	Points	Predicted risk (score)
Kobayashi et al (1)	Japan	2006	528	148	Age≤ 12 mo	1	Low risk(0-2)<br>High risk(≥3)
					illness days≤ 4d	2	
					C-reactive protein≥ 10mg/dL	1	
					aspartate aminotransferase≥ 100IU/L	2	

					platelet≤ 300×10^9/L	1	
					sodium ion concentration≤ 133mmol/L	2	
					% neutrophils≥ 80%	2	
Egami et al (2)	Japan	2006	320	41	Age≤ 6 mo	1	Low risk(0-2) High risk(≥3)
					illness days≤ 4d	1	
					C-reactive protein≥ 8mg/dL	1	
					alanine aminotransferase≥ 80IU/L	2	
					platelet≤ 300×10^9/L	1	
Sano et al (3)	Japan	2007	112	22	C-reactive protein≥ 7mg/dL	1	Low risk(0-2) High risk(≥3)
					total bilirubin≥ 0.9mg/dl	1	
					aspartate aminotransferase≥ 200IU/L	1	
Fu et al (4)	China	2013	1171	211	polymorphous exanthema	1	Low risk(0-3) High risk(≥4)
					changes around the anus	1	
					days of illness at initial treatment≤ 4d	2	
					C-reactive protein≥ 8mg/dL	2	
					% neutrophils≥ 80%	2	
Formosa et al (5)	Taiwan	2016	181	22	lymphadenopathy	1	Low risk(0-2) High risk(≥3)
					% neutrophils≥ 60%	2	
					serum albumin＜ 35g/L	1	
Kawamura et al (6)	Japan	2016	405	85	neutrophil count-to-lymphocyte count ratio≥ 3.83	1	Low risk(0-1) High risk(≥2)
					platelet count-to-lymphocyte count ratio≥ 150	1	
Tang et al (7)	China	2016	910	46	Age≤ 6 mo	2	Low risk(0-2) High risk(≥3)
					Rash	1	
					edema of extremities	1	
					% neutrophils≥ 80%	1	
					serum albumin＜ 35g/L	2	
Yang et al (8)	China	2019	1360	78	C-reactive protein≥ 9mg/dL	3	Low risk(0-5) High risk(≥6)
					% neutrophils≥ 70%	2.5	
					sodium ion concentration＜ 135mmol/L	3	
					serum albumin＜ 35g/L	2.5	
					total bilirubin＞ 20umol/L	5	
Lan et al (9)	China	2018	1655	81	% neutrophils≥ 80%	1	Low risk(0-1) High risk(≥2)
					platelet≤ 278×10^9/L	1	
					total bilirubin≥ 18.5umol/L	1	
					sodium ion concentration≤ 135mmol/L	1	
Liping et al	China	2019	832	120	male	1	

(10)					Age≥ 24 mo	1	Low risk(0-4)<br>High risk(≥5)
					days of illness at initial treatment≤ 5d	2	
					% neutrophils≥ 75%	1	
					hemoglobin concentration≤ 110g/L	1	
					serum albumin＜ 34g/L	2	
					sodium ion concentration≤ 133mmol/L	2	
Wu et al (11)	China	2020	246	31	Age≤ 24 mo	3	Low risk(0-6.5)<br>High risk(≥6.5)
					peripheral neutrophil count≥ 10×10^9/L	3	
					peripheral lymphocyte count≤ 3×10^9/L	3.5	
					peripheral mean platelet volume≥ 10.5fL	3.5	
					serum albumin≤ 37g/L	2.5	

Table 2 Comparisons of the scoring systems between intravenous immunoglobulin non-responsive and responsive KD groups

	IVIG-nonresponsive KD group (n=63)	IVIG-responsive KD group (n=283)	P
Kobayashi et al., 2006 (1) [point, P_{50} (P_{25}, P_{75})]	1.00 (1.00, 2.00)	1.00 (0.00, 2.00)	0.006
Egami et al., 2006 (2) [point, P_{50} (P_{25}, P_{75})]	2.00 (1.00, 4.00)	1.00 (1.00, 3.00)	0.114
Sano et al., 2007 (3) [point, P_{50} (P_{25}, P_{75})]	1.00 (0.00, 1.00)	0.00 (0.00, 1.00)	0.107
Fu et al., 2013 (4) [point, mean±SD]	2.40±2.08	1.90±1.80	0.054
Formosa et al., 2016 (5) [point, P_{50} (P_{25}, P_{75})]	2.00 (1.00, 4.00)	2.00 (0.00, 3.00)	0.171
Kawamura et al., 2016 (6) [point, P_{50} (P_{25}, P_{75})]	0.00 (0.00, 2.00)	0.00 (0.00, 1.00)	0.085

Tang et al., 2016 (7) [point, mean±SD]	2.44±1.64	1.72±1.50	0.001
Yang et al., 2019 (8) [point, P_{50} (P_{25}, P_{75})]	5.50 (2.50, 8.50)	3.00 (0.00, 5.50)	0.001
Lan et al., 2018 (9) [point, P_{50} (P_{25}, P_{75})]	1.00 (0.00, 2.00)	1.00 (0.00, 1.00)	0.006
Liping et al., 2019 (10) [point, P_{50} (P_{25}, P_{75})]	4.00 (2.00, 6.00)	3.00 (2.00, 4.00)	<0.001
Wu et al., 2020 (11) [point, mean±SD]	6.48±2.83	5.21±3.03	0.002

IVIG, intravenous immunoglobulin; KD, Kawasaki disease.

Reference

1. Kobayashi T, Inoue Y, Takeuchi K, Okada Y, Tamura K, Tomomasa T, et al. Prediction of intravenous immunoglobulin unresponsiveness in patients with Kawasaki disease. Circulation. 2006;113(22):2606-12. doi:10.1161/CIRCULATIONAHA.105.592865
2. Egami K, Muta H, Ishii M, Suda K, Sugahara Y, Iemura M, et al. Prediction of resistance to intravenous immunoglobulin treatment in patients with Kawasaki disease. J Pediatr. 2006;149(2):237-40. doi:10.1016/j.jpeds.2006.03.050
3. Sano T, Kurotobi S, Matsuzaki K, Yamamoto T, Maki I, Miki K, et al. Prediction of non-responsiveness to standard high-dose gamma-globulin therapy in patients with acute Kawasaki disease before starting initial treatment. Eur J Pediatr. 2007;166(2):131-7. doi:10.1007/s00431-006-0223-z
4. Fu PP, Du ZD, Pan YS. Novel predictors of intravenous immunoglobulin resistance in Chinese children with Kawasaki disease. Pediatr Infect Dis J. 2013;32(8):e319-23. doi:10.1097/INF.0b013e31828e887f
5. Lin MT, Chang CH, Sun LC, Liu HM, Chang HW, Chen CA, et al. Risk factors and derived formosa score for intravenous immunoglobulin unresponsiveness in Taiwanese children with Kawasaki disease. J Formos Med Assoc. 2016;115(5):350-5. doi:10.1016/j.jfma.2015.03.012
6. Kawamura Y, Takeshita S, Kanai T, Yoshida Y, Nonoyama S. The Combined Usefulness of the Neutrophil-to-Lymphocyte and Platelet-to-Lymphocyte Ratios in Predicting Intravenous Immunoglobulin Resistance with Kawasaki Disease. J Pediatr. 2016;178:281-4.e1. doi:10.1016/j.jpeds.2016.07.035
7. Tang Y, Yan W, Sun L, Huang J, Qian W, Ding Y, et al. Prediction of intravenous immunoglobulin resistance in Kawasaki disease in an East China population. Clin Rheumatol. 2016;35(11):2771-6. doi:10.1007/s10067-016-3370-2
8. Yang S, Song R, Zhang J, Li X, Li C. Predictive tool for intravenous immunoglobulin resistance of Kawasaki disease in Beijing. Arch Dis Child. 2019;104(3):262-7.

doi:10.1136/archdischild-2017-314512
9. Lan X, Jing Z, Lunyu Y, Ling Q, Ying Y, Xiaochun Y. Predictive analysis of intravenous immunoglobulin unresponsive Kawasaki disease. J Clin Pediatr. 2018;36(10):765-71. doi:10.3969/j.issn.1000-3606.2018.10.010
10. LiPing X, Juan G, Yang F, Lan H, Chen C, WeiLi Y, et al. Questioning the establishment of clinical prediction model for intravenous immunoglobulin resistance in children with Kawasaki disease. Chin J Evid Based Pediatr. 2019;14(3):169-75. doi:10.3969/j.issn.1673-5501.2019.03.002
11. Wu S, Liao Y, Sun Y, Zhang CY, Zhang QY, Yan H, et al. Prediction of intravenous immunoglobulin resistance in Kawasaki disease in children. World J Pediatr. 2020;16(6):607-13. doi:10.1007/s12519-020-00348-2
