# Supplementary material for: Risk Factors for Resistance to Intravenous Immunoglobulin Treatment and Coronary Artery Abnormalities in a Chinese Pediatric Population With Kawasaki Disease: A Retrospective Cohort Study
Source: Front Pediatr. 2022 Apr 20;10:812644. doi: 10.3389/fped.2022.812644 (PMC9067160; doi:10.3389/fped.2022.812644)
Supplement: Supplementary file 2 [file Data_Sheet_2.pdf]

Risk Factors for Resistance to Intravenous Immunoglobulin Treatment and Coronary Artery Abnormalities with high-risk Kawasaki Disease based on the Kobayashi score

Over the period of observation, 452 children were admitted to the Pediatrics of First Affiliated Hospital of Guangxi Medical University with a diagnosis of Kawasaki disease (KD). In total, 106 children were excluded from this study, including 53 children who were diagnosed after 10 days of illness, 17 children who received intravenous immunoglobulin (IVIG) or hormone therapy outside the hospital, and 36 with incomplete clinical or laboratory data. Ultimately, 346 children were included in this study. Of them, 63 (18.2%) children had initial IVIG resistance, and the Kobayashi score (1) and five Chinese scoring system scores (Tang et al.(2), Yang et al.(3), Lan et al.(4), Liping et al.(5), and Wu et al.(6)) were higher in the IVIG non-responsive KD group than in the IVIG responsive KD group, and the differences were statistically significant (all, $P<0.05$). The area under the receiver operating characteristic (ROC) curves for the Xie Liping risk scoring system was the largest, with an area of 0.650 (95% confidence interval [CI]: 0.571–0.729), while the Kobayashi score with an area of 0.608 (95% CI: 0.527–0.688). Eighty-seven patients were diagnosed with high-risk KD using the Xie Liping risk scoring system and 62 patients were diagnosed with high-risk KD using the Kobayashi score, and an overlap was observed between the two groups for a total of 39 patients.

1 Intragroup comparisons between the high-risk KD groups to analyze the risk factors for high-risk KD

1.1 Analysis of risk factors for IVIG resistance in high-risk KD

1.1.1 Comparisons of the clinical characteristics

Sixty-two hospitalized children were diagnosed with high-risk KD according to the scoring system (Kobayashi score of ≥ 4 points). Of these, 20 patients with IVIG resistance were enrolled in the IVIG non-responsive sub-group, and 7 (35.0%) of them had used corticosteroid therapy in addition to the second dose of gammaglobulin therapy. The remaining 42 patients were enrolled in the IVIG responsive sub-group. The sum of scores based on the Kobayashi scoring system, and the proportion of days of illness at primary treatment (< 5 days), were higher in the IVIG non-responsive sub-group than in the IVIG responsive sub-group with statistically significant differences (both, $P<0.05$). However, there were no statistically significant differences between the sub-groups with respect to age, sex, body mass index (BMI), pediatric sequential organ failure assessment (pSOFA) score, fever duration before admission, and the incidence of incomplete KD or coronary artery aneurysm (CAA) (all, $P>0.05$). In terms of laboratory indicators, the IVIG non-responsive sub-group

showed higher values of the serum total bilirubin (TSB) level and TSB-to-albumin ratio (B/A ratio) and lower values of the serum sodium and albumin than the IVIG responsive sub-group, and the differences were statistically significant (all, P<0.05), as shown in Table 1.

Table 1 Comparison of the baseline characteristics between the intravenous immunoglobulin non-responsive and responsive sub-groups

	Total (n=62)	IVIG non-responsive sub-group (n=20)	IVIG responsive sub-group (n=42)	P-value
Age [month, P_{50} (P_{25}, P_{75})]	24.50 (11.75, 49.25)	24.50 (13.25, 62.25)	24.50 (11.00, 38.25)	0.527
<1 year [n(%)]	8 (12.9)	4 (20.0)	4 (9.5)	0.456
Male [n(%)]	44 (71.0)	14 (70.0)	30 (71.4)	0.908
BMI [kg/m^2, P_{50} (P_{25}, P_{75})]	15.73 (14.51, 17.15)	15.58 (14.06, 17.31)	15.79 (14.75, 17.02)	0.821
pSOFA score [point, mean±SD]	1.19±1.84	1.60±2.44	1.00±1.47	0.232
Fever duration before admission [day, mean±SD]	5.52±2.84	5.65±2.80	5.45±2.89	0.800
Days of illness at primary treatment [day, P_{50} (P_{25}, P_{75})]	5.00 (4.00, 7.00)	5.00 (3.25, 6.50)	6.00 (4.00, 8.00)	0.044
≤4 days [n(%)]	22 (35.5)	9 (45.0)	13 (31.0)	0.280

≤5 days [n(%)]	33 (53.2)	15 (75.0)	18 (42.9)	0.018
Incomplete KD [n(%)]	32 (51.6)	11 (55.0)	21 (50.0)	0.713
CAA [n(%)]	19 (30.6)	5 (25.0)	14 (33.3)	0.506
Score (kobayashi) (1) [point, P_{50} (P_{25}, P_{75})]	5.00 (4.00, 6.00)	5.00 (4.25, 7.00)	4.00 (4.00, 5.00)	0.016
White blood cell count [$\times10^9$/L, ref. 5–12$\times10^9$/L, mean±SD]	13.89±6.10	12.25±5.72	14.67±6.19	0.147
Neutrophils count [$\times10^9$/L, ref. 1.8–6.3$\times10^9$/L, mean±SD]	10.36±5.64	9.64±4.53	10.70±6.12	0.493
≥80% [n(%)]	31 (50.0)	12 (60.0)	19 (45.2)	0.277
NLR [mean±SD]	6.27±4.78	7.65±5.63	5.62±4.23	0.117
Hemoglobin [g/L, ref. 120–160 g/L, mean±SD]	106.18±15.16	108.14±16.18	105.25±14.77	0.487
Platelet count [$\times10^{12}$/L, ref.125–350$\times10^9$/L, mean±SD]	274.12±110.89	251.10±103.89	285.08±113.63	0.263
PLR [mean±SD]	167.11±129.74	203.49±142.47	149.78±121.18	0.129

CRP [mg/L, ref. 0–10 mg/L, P_{50} (P_{25}, P_{75})]	101.10 (57.43,183.47)	106.23 (56.92, 151.50)	100.95 (56.32, 192.00)	0.820
Sodium [mmol/L, ref. 137–147 mmol/L, P_{50} (P_{25}, P_{75})]	132.95 (131.18,137.10)	132.50 (130.53, 133.45)	134.45 (131.88, 137.55)	0.018
≤133 mmol/L [n(%)]	32 (51.6)	15 (75.0)	17 (40.5)	0.011
ALT [U/L, ref. 7–45 U/L, mean±SD]	98.66±93.01	113.44±62.08	91.62±104.54	0.392
AST [U/L, ref. 13–40 U/L, mean±SD]	82.92±112.29	68.86±38.96	89.62±133.84	0.501
Total bilirubin [µmol/L, ref. 3.4–20.5 µmol/L, P_{50} (P_{25}, P_{75})]	8.00 (4.15, 17.13)	16.15 (8.35, 29.83)	6.50 (3.38, 12.25)	0.002
Albumin [g/L, ref. 40–55 g/L, mean±SD]	34.55±5.06	32.54±4.48	35.51±5.09	0.029
≤34 g/L [n(%)]	25 (40.3)	12 (60.0)	13 (31.0)	0.029
B/A ratio [P_{50} (P_{25}, P_{75})]	0.25 (0.11, 0.61)	0.47 (0.26, 0.77)	0.20 (0.10, 0.36)	0.001
CLI [P_{50} (P_{25}, P_{75})]	3.22 (1.72, 4.94)	3.36 (1.85, 4.57)	2.96 (1.59, 5.08)	0.964

IVIG, intravenous immunoglobulin; BMI, body mass index; pSOFA, pediatric sequential organ failure assessment; KD, Kawasaki disease; CAA, coronary artery aneurysm; NLR, neutrophil-to-lymphocyte count ratio; PLR, platelet-to-lymphocyte count ratio; CRP, C-reactive protein; ALT, alanine aminotransferase; AST, aspartate

aminotransferase; B/A ratio, total bilirubin-to-albumin ratio; CLI, capillary leakage index.

1.1.2 Results of the multi-factor logistic analysis

To determine the relative effect of each risk factor for IVIG resistance in high-risk KD, we performed a logistic regression analysis, which revealed that high-risk KD with IVIG resistance was significantly associated with four baseline laboratory variables (serum levels of TSB, sodium, albumin, and B/A ratio) and two clinical characteristics (days of illness at primary treatment and the Kobayashi score). These variables plus the C-reactive protein (CRP) level, platelet (PLT) count, age <1 year and the percentage of neutrophils≥80%, all of which were previously reported as risk factors for IVIG resistance (1, 3, 7-9), but not the Kobayashi score, TSB or albumin level, were included in the logistic regression analysis (Table 2). The B/A ratio, calculated as the TSB level divided by the albumin level, was included in the multivariable analysis instead of the two separate indicators; the Kobayashi score was also excluded from the multivariable analysis because the variables included in this risk score, such as days of illness at primary treatment, the serum sodium level, age, the percentage of neutrophils, and PLT count, were included in the multivariable model. The B/A ratio, days of illness at primary treatment and serum sodium were significant independent predictor of IVIG resistance in high-risk KD.

Table 2 Results of logistic regression analyses of intravenous immunoglobulin resistance

Characteristic	Univariable		Multivariable	
	Odds ratio (95% CI)	*P*-value	Odds ratio (95% CI)	*P*-value
Days of illness at primary treatment	0.766 (0.584–1.005)	0.055	0.568 (0.349–0.922)	0.022
Sodium	0.817 (0.697–0.957)	0.012	0.746 (0.588–0.946)	0.016
B/A ratio	4.073 (1.249–13.284)	0.020	10.336 (1.240–86.126)	0.031
Neutrophils count ≥80%	1.816 (0.616–5.355)	0.280	1.728 (0.340–8.788)	0.510
CRP	0.999 (0.990–1.007)	0.763	0.995 (0.981–1.009)	0.497

Platelet count	0.997 (0.992–1.002)	0.261	0.997 (0.991–1.004)	0.411
<1 year	0.421 (0.094–1.895)	0.260	0.125 (0.013–1.187)	0.125

CI, confidence interval; B/A ratio, total bilirubin-to-albumin ratio; CRP, C-reactive protein.

1.2 Analysis of risk factors for CAA in high-risk KD

Although there were 62 patients in the high-risk group, only 19 (30.6%) of these patients had CAA, and age and the incidence of incomplete KD were lower in the CAA sub-group than in the non-CAA sub-group with statistically significant differences (both, $P<0.05$). However, there were no statistically significant differences between the sub-groups with respect to sex, BMI, pSOFA score, fever duration before admission, days of illness at primary treatment, the incidence of IVIG resistance and the sum of scores based on the Kobayashi scoring system (all, $P>0.05$), as shown in Table 3. The univariable analysis identified only one laboratory index, the PLT count, which, together with the Z score of the coronary artery internal diameter in the acute phase of high-risk KD, was significantly associated with CAA development. Multivariable analysis revealed that only the Z score of the left main coronary artery internal diameter were significant independent predictors of CAA development, and this difference remained significant when corrected for age and sex (Table 4).

Table 3 Comparisons of the baseline characteristics between the coronary artery aneurysm and non-coronary artery aneurysm sub-groups

	Total (n=62)	CAA sub-group (n=19)	Non-CAA sub-group (n=43)	*P*-value
Age [month, mean±SD]	32.85±26.89	19.58±20.14	38.72±27.59	0.009
<1 year [n(%)]	8 (12.9)	5 (26.3)	3 (7.0)	0.092
Male [n(%)]	44 (71.0)	13 (68.4)	31 (72.1)	0.769
BMI [kg/m^2, mean±SD]	15.91±1.79	16.35±1.70	15.72±1.81	0.200

pSOFA score [point, P_{50} (P_{25}, P_{75})]	1.00 (0.00, 2.00)	1.00 (0.00, 3.00)	1.00 (0.00, 1.00)	0.206
Fever duration before admission [day, mean±SD]	5.52±2.84	5.95±3.98	5.33±2.19	0.431
Days of illness at primary treatment [day, P_{50} (P_{25}, P_{75})]	5.00 (4.00, 7.00)	7.00 (3.00, 9.00)	5.00 (4.00, 7.00)	0.396
≤4 days [n(%)]	22 (35.5)	8 (42.1)	14 (32.6)	0.469
≤5 days [n(%)]	33 (53.2)	8 (42.1)	25 (58.1)	0.243
Incomplete KD [n(%)]	32 (51.6)	5 (26.3)	27 (62.8)	0.008
IVIG resistance [n(%)]	20 (32.3)	5 (26.3)	15 (34.9)	0.506
Score (kobayashi) (1) [point, mean±SD]	5.03±1.24	4.84±1.34	5.12±1.20	0.427
White blood cell count [$\times10^9$/L, ref. 5–12$\times10^9$/L, P_{50} (P_{25}, P_{75})]	13.00 (9.20, 17.88)	12.50 (7.28, 21.98)	13.95 (9.63, 17.35)	0.743
Neutrophils count [$\times10^9$/L, ref.1.8–6.3$\times10^9$/L, P_{50} (P_{25}, P_{75})]	10.19 (6.43, 15.13)	7.11 (4.24, 16.51)	10.53 (7.21, 14.68)	0.459
≥80% [n(%)]	31 (50.0)	6 (31.6)	25 (58.1)	0.054
NLR [mean±SD]	6.27±4.78	5.54±4.76	6.60±4.80	0.428

Hemoglobin [g/L, ref. 120–160 g/L, mean±SD]	106.18±15.16	102.78±17.87	107.68±13.77	0.244
Platelet count [$\times10^{12}$/L, ref. 125–350$\times10^{9}$/L, mean±SD]	274.12±110.89	231.49±98.74	292.95±111.79	0.043
PLR [mean±SD]	167.11±129.74	129.08±126.09	183.91±129.19	0.126
CRP [mg/L, ref. 0–10 mg/L, mean±SD]	111.69±62.76	89.08±63.46	121.67±60.50	0.059
Sodium [mmol/L, ref. 137–147 mmol/L, mean±SD]	133.96±4.25	134.75±4.93	133.61±3.92	0.333
≤133 mmol/L [n(%)]	32 (51.6)	8 (42.1)	24 (55.8)	0.319
ALT [U/L, ref. 7–45 U/L, P_{50} (P_{25}, P_{75})]	82.00 (24.25, 133.00)	54.00 (25.00, 96.00)	87.00 (21.00,151.00)	0.179
AST [U/L, ref. 13–40 U/L, mean±SD]	82.92±112.29	83.79±95.27	82.54±120.09	0.968
Total bilirubin [μmol/L, ref. 3.4–20.5 μmol/L, mean±SD]	15.96±19.18	13.07±16.30	17.23±20.37	0.436
Albumin [g/L, ref. 40–55 g/L, P_{50} (P_{25}, P_{75})]	34.55 (30.88, 37.93)	34.80 (29.20, 41.10)	34.40 (31.70,37.30)	0.927
≤34 g/L [n(%)]	25 (40.3)	8 (42.1)	17 (39.5)	0.849

B/A ratio [mean±SD]	0.49±0.66	0.44±0.67	0.52±0.66	0.655
CLI [mean±SD]	3.34±1.93	2.88±2.24	3.55±1.77	0.210
Z score of coronary artery internal diameter				
Left main coronary artery [mean±SD]	2.46±1.23	3.74±0.99	1.90±0.85	<0.001
Right coronary artery [mean±SD]	2.02±1.36	3.13±1.17	1.54±1.14	<0.001

CAA, coronary artery aneurysm; BMI, body mass index; pSOFA, pediatric sequential organ failure assessment; KD, Kawasaki disease; IVIG, intravenous immunoglobulin; NLR, neutrophil-to-lymphocyte count ratio; PLR, platelet-to-lymphocyte count ratio; CRP, C-reactive protein; ALT, alanine aminotransferase; AST, aspartate aminotransferase; B/A ratio, total bilirubin-to-albumin ratio; CLI, capillary leakage index.

Table 4　Results of logistic regression analyses of coronary artery aneurysm

Characteristic	Univariable		Multivariable		Adjust#	
	Odds ratio (95% CI)	*P*-value	Odds ratio (95% CI)	*P*-value	Odds ratio (95% CI)	*P*-value
Age	0.960 (0.928—0.993)	0.017	0.970 (0.914—1.029)	0.309	0.968 (0.914—1.026)	0.274
Incomplete KD	0.212 (0.064—0.698)	0.011	0.290 (0.037—2.307)	0.242	0.272 (0.033—2.254)	0.227
Platelet count	0.994 (0.989—1.000)	0.050	0.994 (0.985—1.003)	0.179	0.994 (0.986—1.003)	0.200
Z score of left main coronary artery	22.917 (4.014—130.832)	<0.001	17.071 (1.800—161.920)	0.013	19.097 (1.871—194.902)	0.013

Z score of right coronary artery	3.397 (1.716—6.726)	<0.001	1.724 (0.662—4.487)	0.264	1.737 (0.656—4.598)	0.266

#indicates a significant relationship after correction for sex.

CI, confidence interval; KD, Kawasaki disease.

References

1. Kobayashi T, Inoue Y, Takeuchi K, Okada Y, Tamura K, Tomomasa T, et al. Prediction of intravenous immunoglobulin unresponsiveness in patients with Kawasaki disease. Circulation. 2006;113(22):2606-12. doi:10.1161/CIRCULATIONAHA.105.592865
2. Tang Y, Yan W, Sun L, Huang J, Qian W, Ding Y, et al. Prediction of intravenous immunoglobulin resistance in Kawasaki disease in an East China population. Clin Rheumatol. 2016;35(11):2771-6. doi:10.1007/s10067-016-3370-2
3. Yang S, Song R, Zhang J, Li X, Li C. Predictive tool for intravenous immunoglobulin resistance of Kawasaki disease in Beijing. Arch Dis Child. 2019;104(3):262-7. doi:10.1136/archdischild-2017-314512
4. Lan X, Jing Z, Lunyu Y, Ling Q, Ying Y, Xiaochun Y. Predictive analysis of intravenous immunoglobulin unresponsive Kawasaki disease. J Clin Pediatr. 2018;36(10):765-71. doi:10.3969/j.issn.1000-3606.2018.10.010
5. LiPing X, Juan G, Yang F, Lan H, Chen C, WeiLi Y, et al. Questioning the establishment of clinical prediction model for intravenous immunoglobulin resistance in children with Kawasaki disease. Chin J Evid Based Pediatr. 2019;14(3):169-75. doi:10.3969/j.issn.1673-5501.2019.03.002
6. Wu S, Liao Y, Sun Y, Zhang CY, Zhang QY, Yan H, et al. Prediction of intravenous immunoglobulin resistance in Kawasaki disease in children. World J Pediatr. 2020;16(6):607-13. doi:10.1007/s12519-020-00348-2
7. Egami K, Muta H, Ishii M, Suda K, Sugahara Y, Iemura M, et al. Prediction of resistance to intravenous immunoglobulin treatment in patients with Kawasaki disease. J Pediatr. 2006;149(2):237-40. doi:10.1016/j.jpeds.2006.03.050
8. Fu PP, Du ZD, Pan YS. Novel predictors of intravenous immunoglobulin resistance in Chinese children with Kawasaki disease. Pediatr Infect Dis J. 2013;32(8):e319-23. doi:10.1097/INF.0b013e31828e887f
9. Miyata K, Miura M, Kaneko T, Morikawa Y, Sakakibara H, Matsushima T, et al. Risk Factors of Coronary Artery Abnormalities and Resistance to Intravenous Immunoglobulin Plus Corticosteroid Therapy in Severe Kawasaki Disease: An Analysis of Post RAISE. Circ Cardiovasc Qual Outcomes. 2021;14(2):e007191. doi:10.1161/CIRCOUTCOMES.120.007191
